# Psychiatrists’ attitudes to professional boundaries concerning spirituality and religion: mixed-methods study

**DOI:** 10.1192/bjb.2023.66

**Published:** 2024-08

**Authors:** Rob Poole, Christopher C. H. Cook, Robert Song, Catherine A. Robinson

**Affiliations:** 1 Bangor University, Bangor, UK; 2 Durham University, Durham, UK; 3 University of Manchester, Manchester, UK

**Keywords:** Qualitative research, ethics, clinical governance, transcultural psychiatry, psychosocial interventions

## Abstract

**Aims and method:**

Calls for the integration of spirituality into psychiatric practice have raised concerns about boundary violations. We sought to develop a method to capture psychiatrists’ attitudes to professional boundaries and spirituality, explore consensus and understand what factors are considered. Case vignettes were developed, tested and refined. Three vignettes were presented to 80 mental health professionals (53% said they were psychiatrists; 39% did not identify their professional status). Participants recorded their reactions to the vignettes. Four researchers categorised these as identifying boundary violations or not and analysed the factors considered.

**Results:**

In 90% of cases, at least three of the four researchers agreed on classification (boundary violation; possible boundary violation; no boundary violation). Participants’ opinion about boundary violations was heterogeneous. There was consensus that psychiatrists should not proselytise in clinical settings. Reasoning emphasised pragmatic concerns. Few participants mentioned their religious beliefs. Equivocation was common.

**Clinical implications:**

Mental health professionals seem unsure about professional boundaries concerning religion and spirituality in psychiatric practice.

There has been significant interest in the relationship between mental health, spirituality and religion over the past two decades. There is a substantial literature on the subject,^1^ although the strength of the findings has been contested.^2^ Calls for integration of spirituality into clinical practice^3^ have led to concern about the implications for the maintenance of professional boundaries in therapeutic relationships.^4^ Alongside these controversies, mindfulness-based therapies,^5^ which derive from Buddhist spiritual practice, are increasingly offered by mainstream services. There is controversy over the extent to which mindfulness can be secularised, and there is evidence of enduring alterations to patients’ spirituality following such therapies.^6^

The Royal College of Psychiatrists^7^ and the World Psychiatric Association^8^ have issued position statements that are broadly supportive of the integration of spirituality into clinical practice. Although both documents warn of a duty to avoid abuse, neither is prescriptive regarding appropriate boundaries. There is no empirical evidence on psychiatrists’ attitudes. There is broad consensus over sexual and financial boundaries, but this is not the case with spirituality or religion.

Two of us (R.P. and C.C.H.C.) have taken different stances in the debate.^9^ We agree that professional boundaries are important with respect to spirituality and religion. We disagree about a range of other issues. R.P. has argued that the introduction of religion into clinical practice under the label of ‘spirituality’ creates risks to patients including: abuses of power (for example, proselytisation); imposition of faith-based values (for example, hostility to homosexuality); and alienation of patients whose beliefs differ from the clinician's.^4^ There is a need for evidence about the attitudes of psychiatrists and others involved in mental health services prior to exploring patients’ attitudes, as the former are likely to be the critical factor determining whether psychiatrists use spirituality in their practice.

## Method

### Development

Given the impossibility of neutrality about religion, joint design and conduct of the study by researchers with different stances offers protection against researcher bias. Our research team consisted of two Christians and two atheists; two of us are psychiatrists, one is a social scientist and one is a medical ethicist.

Too little is known about professional and patient attitudes to allow quantitative research. Qualitative research methods are appropriate for exploratory studies, especially when addressing issues of medical culture, attitudes and the application of values.^10^

We were unable to identify an existing method to explore the questions of interest, which were:

- Is there a consensus among psychiatrists (and others with an interest in mental health) about what should be regarded as actual or potential boundary breaches concerning religion in clinical situations?
- What factors do psychiatrists (and others with an interest in mental health) take into account in making these judgements?

We therefore developed a bespoke method.

We held workshops involving mental health professionals as research participants. They were offered clinical vignettes that described scenarios involving religion or spirituality in clinical practice. Participants were asked to record their immediate response to the scenario. They were told that we were interested in professional boundaries, but the concept of ‘boundaries’ was not defined. Participants were not asked to answer a specific or categorical question. We then invited them to discuss the vignette.

Vignettes were developed to reflect realistic clinical situations without breaching confidentiality. Draft vignettes were provided by the executive committee of the Royal College of Psychiatrists’ Spirituality and Psychiatry Special Interest Group and members of Bangor University's Centre for Mental Health and Society. They ranged between 25 and 300 words in length. They were edited to remove clues to the authors’ opinions and to make them as simple as possible without losing nuance. The edited versions sought to simply describe a clinical situation, which sometimes included responses of the patient or clinical staff.

Three field trial workshops about religion and professional boundaries were conducted with participants from a variety of professional backgrounds: UK senior psychiatric trainees; Welsh mental health professionals and managers; and Finnish social work students. Some vignettes generated more discussion than others, and simplifications and clarifications were made in the light of experience.

Different methods of capturing participants’ responses were trialled, including small-group discussion with note-keeping and ‘sticky notes’ boards. Participants were keen to discuss ethical issues, and some appeared to change their minds as a result of discussion. Discussions were particularly helpful in demonstrating participants’ reasoning and ethical stance.

### The study: programmed workshop

The three vignettes that generated the most discussion in the field trials (see under Results) were selected for use in a programmed workshop at the Royal College of Psychiatrists’ International Congress in Birmingham in June 2015. Participation in the workshop was not conditional on consent to the use of personal responses in this research.

The purpose of the workshop was explained to participants. Each vignette was projected on a large screen, read out by the facilitator and provided as a printed copy. Participants were asked to write down their immediate thoughts about the vignette. The findings below are entirely based on these immediate responses.

The group then engaged in discussion, which exposed participants to a range of responses. The main points were recorded by a member of the research team. At the end of the discussions, each lasting about 20 min, participants were asked to write down whether their views had changed and further reflections. These data are not analysed here. At the end of the session, participants were asked to add their job title to their response sheet.

All responses were handwritten. Participants were not asked to decide whether there was boundary violation within the vignette. Some participants did not respond to all three vignettes.

### Consent

The information sheet stated that participation was voluntary and that participants were free to withdraw. Participants could choose not to hand in their written responses. Written responses were anonymous and were not linked to individuals. The information sheet stated that anonymised findings would be used in research studies and in academic papers and that, by completing and handing in written responses, participants were consenting to take part in the study. The information sheet was read at the start of the workshop.

### Ethical approval

Ethical approval was granted by the Research Ethics Committee of Bangor University College of Business, Law, Education and Social Sciences.

### Method of analysis

Responses were subject to two types of analysis. First, we took a deductive approach to assess the extent to which respondents seemed to think that the vignette described a boundary violation, using a simple coding framework. Second, we took an inductive approach to analyse comments about boundary violations to capture participants’ reasoning and what ethical considerations they took into account.

### Assessment of participants’ perceptions

Responses were often very brief. To assess whether they could be interpreted with consistency, every response was read by each of the four researchers (C.C.H.C., R.P., C.A.R., R.S.). They independently coded the responses against the following categories:

- a possible boundary violation
- a definite boundary violation
- no boundary violation.

A small number of the responses were illegible or the participant's opinion could not be discerned.

The researchers met as a group to discuss any responses on which they disagreed about the participants’ opinion. This allowed the research team to understand why disagreement had occurred.

## Results

There were 80 participants in the workshop, 68 (85%) of whom chose to contribute to the research. They generated a total of 203 responses across the three vignettes. The professional background of participants is set out in Table 1.

**Table 1:** Declared professional designation of participants (*n* = 80)

Declared designation	Participants, *n* (%)
Consultant psychiatrist	23 (29)
Career grade psychiatrist	5 (6)
Psychiatrist in training	14 (17)
Psychiatrist (not otherwise specified)	1 (1)
Medical student/foundation doctor	3 (4)
Non-medical researcher	3 (4)
No designation declared	31 (39)

### Researcher agreement about participants’ perceptions

From a total of 202 responses, all four researchers agreed in 113 cases (55.9%). In other words, the researchers completely agreed that the participant was indicating that the vignette involved an actual boundary violation, a potential boundary violation or no boundary violation.

In a further 63 cases (31.2%), three researchers agreed and one differed, but there was not complete disagreement (i.e. between ‘boundary violation’ and ‘no boundary violation’; in other words, at least one researcher thought that the participant was expressing a view that there was a potential boundary violation).

In 21 cases (10.4%) the researchers were divided into two groups of two, where one group's assessment was that the participant thought that there was a potential boundary violation. In only 5 cases (2.5%) was there a wide disagreement among the researchers.

No researcher was significantly more likely to be in a minority of one than any other; the number of instances for each researcher ranged from 10 to 15.

To summarise, there was good agreement among the four researchers about the content of the responses, at least three out of the four agreeing about 176 (87.1%) of the cases, a lack of consensus for 21 cases (10.4%) and complete disagreement in only 5 cases (2.5%).

The next section describes how those 176 judgements are distributed for each of the three vignettes.

### Analysis of participants’ judgements

#### Vignette 1

A small number of participants (4) did not express any view about boundary violation in Vignette 1 (Box 1). The largest group (28) felt that there was a potential boundary violation, with many participants basing this decision on the particular circumstances, for example:

‘It depends in what situation this occurred. Is the psychiatrist enforcing their view on the patient or did the patient initiate the discussion?’

Box 1 Vignette 1

On two occasions a junior psychiatrist is seen by nursing staff in earnest conversation and reading the Bible with a psychiatric in-patient. The nurse is concerned and informs the consultant.

Similarly:

‘If junior is practising religion on patient it may be crossing boundaries.’

A smaller number of participants (19) were more categorical and suggested that there was a definite boundary violation. One participant suggested that this was outside the doctor's role:

‘Inappropriate as patient is vulnerable and the doctor may be imposing their belief on them. Even if they showed religious belief it is not the role of a doctor [ … ].’

Another participant highlighted the potential to create disparities:

‘[ … ] This practice may be seen as unfair and exclude patients of other religions.’

The smallest group (8) felt there was no boundary violation, with one participant suggesting it was acceptable if it helped the patient's treatment.

#### Vignette 2

Most (36) participants felt that there was no boundary violation in Vignette 2 (Box 2) and that the psychiatrist and the priest has acted appropriately to reduce the patient's distress. Some participants referred to the Mental Health Act to support this view:

‘I think no boundaries have been crossed here as reading the Bible was part of her delusion and distress. She was clearly psychotic and because of her delusion the Bible was causing her distress. Under the MH Act [the] Bible was causing her distress.’

Box 2 Vignette 2

A 25-year-old African woman was admitted to a medical ward with malnutrition. She refused to eat and the possibility of a diagnosis of anorexia was considered. Assessment revealed a woman reading the Bible, very reluctant to engage with anyone who was not a Christian. She described a message from the Bible that God wanted her to be pure and live on the Holy Spirit alone. This precluded eating and drinking and God needed to test her faith. She accepted IV hydration, but pulled out the line after God spoke to her in the night. Collateral information from parents revealed a strong family history of schizophrenia and a reclusive existence since developing paranoid ideas in the workplace 1 year previously. She had been collecting faeces in saucepans at home and thought her neighbour was poisoning her. Her priest attended and confided that he had told her that she should eat. On the recommendation of the priest and family the staff took away her Bible from her until she got better, as it was reinforcing her delusions. She was treated under the Mental Health Act for schizophrenia and necessary physical treatment was provided under the Mental Capacity Act.

Other participants (14) were less certain and suggested that there was a potential boundary violation:

‘Taking away her Bible, however, may represent and infringement of civil liberty. What would be interesting would be to know if she was able to regain her faith, minus delusions, after recovery or not.’

One participant queried whether consultation with the priest had slowed down treatment decisions, questioning the relevance of removing the Bible:

‘Good that the priest attended but what would have been the outcome if he had not attended? Team would have sought psychiatric opinion quickly and initiated [treatment] with antipsychotic medication earlier. I'm not sure if taking the Bible away was really appropriate if she really wanted it.’

For a small number of participants (5), the actions of the clinician involved a definite boundary violation. For some this was about involving the priest in any way and for others it was about removing the Bible.

#### Vignette 3

Similar numbers of participants felt that there was no boundary violation (12) compared with those who felt that there was a potential boundary violation (9) in vignette 3 (Box 3). Nearly two-thirds (32) of participants felt that there was a definite boundary violation. Interestingly, many of the participants drew attention to the apparent beneficial effects of the approach, even among those who were categorical in seeing this vignette as a definite boundary violation:

‘Totally unacceptable, though the outcome may have been good, the psychiatrist was not in this role (e.g. Spiritualist) and not offering psychiatric care that the patient would be expected to receive. What would the defence be if the outcome had been negative for this intervention. This is an abuse of the power differential here.’

Box 3 Vignette 3

A young man presented feeling ‘not himself’ since the suicide of a good friend several months before. She had died in the patient's home while he was away on holiday. Antidepressant medication had been tried but he was ‘still not feeling himself’. On a hunch the psychiatrist asked him if he had the feeling of someone else. He replied he hadn't wanted to mention it in case it sounded mad, but every time he went into the house he could feel the presence of his friend right there in the room with him. The psychiatrist then asked the patient if he would like to try ‘speaking’ with the friend to find out what was wrong. He was keen to do this, so he was asked to close his eyes, tune in to her and sense what she might be feeling. The patient found himself voicing his friend's deep regret at having taken her life, saying ‘If only I had known what I know now. I was facing the biggest challenge of my life and I went and messed it up. I feel even worse than I did before’. The psychiatrist explained to the ‘sensed’ friend that her continuing presence was distressing his patient and was doing nothing to help her either. He assured her that other opportunities would be given her and she sounded very relieved. After apologising to the patient, she agreed that she was ready to move on. The psychiatrist asked her to look for ‘the light’ and go there. She exclaimed ‘Yes, I can see it’ and left at once. From this moment, the patient felt the burden of oppression lift from him and it did not return.

Some participants expressed strong feelings about this vignette, suggesting it involved a clear boundary violation:

‘I do not think the psychiatrist's actions were acceptable. It was unprofessional, not a valid psychiatric technique and fed into the patients delusions about the “spirit” he experienced.’

Those who felt that there was no boundary violation justified this on the basis of the apparent success of the approach, while acknowledging some reservations:

‘The psychiatrist was correct in his/her approach with their patient. While the role-play which occurred was bizarre, it seemed to provide great relief to the patient. This may have crossed a line when encouraging them into “the light”, assuming an afterlife, however this appeared to be where the patient wanted this session to go. There is also a question regarding the evidence base for the treatment.’

A small number of participants were unquestioning:

‘The psychiatrist was pursuing a “safe space” to explore the thinking of the patient and also allowing the potential of therapeutic narrative that enabled the patient to “move through” the period of not “feeling himself”.’

## Discussion

This study does not explore the nature of professional boundaries, nor where they should lie. It is concerned with opinions of participants on the basis of their existing conceptualisation of these matters. Our findings suggest that opinion about boundary violations with regard to religion and spirituality in psychiatric practice is highly heterogeneous, with little evidence of an overall consensus as to where boundaries lie, even in relatively uncomplicated scenarios such as Vignette 1. There does appear to be clear consensus that psychiatrists should not proselytise their own beliefs in clinical settings, but there are differences as to what behaviour might be regarded as proselytisation, as seen in differences over Vignette 3.

Responses seemed to draw on both first principles and pragmatic justifications; in other words, some rules were stated as absolute (e.g. doctors should not proselytise to their patients), but some unusual behaviour by doctors was said to be justified because there was perceived to have been a positive outcome. This is perhaps to be expected, as the tension between inviolable rules and a pragmatic focus on outcomes is common throughout medical practice. Overall, our participants showed a greater tendency to emphasise outcomes rather than principles. There was often rationalisation of unusual behaviour of doctors in the vignettes by reference to a positive outcome. Very few responses mentioned the participants’ own religious beliefs (or lack of them) in interpreting the vignettes.

Our study has shown that participants were often uncertain about potential boundary violations, with many individual responses describing conflicting perspectives on a vignette without taking a firm stance. Our findings do not provide particular support either for those who advocate or those who oppose greater integration of spirituality and religion into psychiatric practice.

### Strengths, limitations and future research

The method that we have developed and piloted has allowed a research team with strong and conflicting views to work together constructively to generate findings that we all accept are valid. Although our findings are inconclusive, we believe that it will be more productive to build an empirical evidence base in this way than to pursue a polarised and repetitive debate through rhetoric based on selective citation. We recommend that future research teams should embrace a diversity of opinion in this way.

Our study has some limitations. The participants were self-selected conference attenders who were interested in the topic, and are therefore unlikely to be representative of psychiatrists in general. The decision whether to submit written responses for analysis was made after group discussion. We cannot exclude the possibility that those with minority opinions chose not to submit.

It would be fruitful to replicate our work with vignettes that explore different scenarios. This might usefully include vignettes exploring different boundaries, for example those concerning sexual behaviour, in order to understand more about how the participants conceptualise boundaries, and their violation, in general.

## About the authors

**Rob Poole** is Professor of Social Psychiatry in the Centre for Mental Health and Society, Bangor University, Bangor, UK. **Christopher C.H. Cook** is Emeritus Professor of Spirituality, Theology and Health in the Institute of Medical Humanities at Durham University, UK. **Robert Song** is Professor of Theological Ethics in the Department of Theology and Religion at Durham University, UK. **Catherine A. Robinson** is Professor of Social Care Research and Director of Social Care and Society in the School of Health Sciences at the University of Manchester, UK.

## Data Availability

The data that support the findings of this study are available from the corresponding author, R.P., upon reasonable request.
